# Poster Session I - A182 REVERSE HEPATITIS B SEROCONVERSION WITHOUT IMMUNOSUPPRESION: A CASE REPORT

**DOI:** 10.1093/jcag/gwaf042.182

**Published:** 2026-02-13

**Authors:** K Zhu, E Wu, B R Tam, E Yoshida, P Kwan

**Affiliations:** Gastroenterology, The University of British Columbia Faculty of Medicine, Vancouver, BC, Canada; The University of British Columbia Faculty of Medicine, Vancouver, BC, Canada; Gastroenterology, The University of British Columbia Faculty of Medicine, Vancouver, BC, Canada; Gastroenterology, The University of British Columbia Faculty of Medicine, Vancouver, BC, Canada; Gastroenterology, The University of British Columbia Faculty of Medicine, Vancouver, BC, Canada

## Abstract

**Background:**

Hepatitis B virus (HBV) and hepatitis C virus (HCV) are leading causes of chronic liver disease worldwide. HBV-HCV coinfection is associated with more severe outcomes, including accelerated cirrhosis, and hepatocellular carcinoma. After apparent clearance of HBV, the presence of hepatitis B surface antibody (anti-HBs) generally signifies long-term immunity. Rarely, HBV can reactivate, leading to reappearance of hepatitis B surface antigen (HBsAg), a phenomenon termed reverse seroconversion. This typically occurs in the context of immunosuppression, but spontaneous reactivation without an identifiable trigger is extremely uncommon.

**Aims:**

To present a case of hepatitis B reverse seroconversion in a patient with previously resolved HBV and sustained HCV response.

**Methods:**

Case report

**Results:**

We report an 88-year-old woman with a history of HCV genotype 1b infection, successfully treated with direct-acting antivirals in 2014. At that time, her HBV serology confirmed resolved infection, with negative HBsAg, positive core antibody, and protective anti-HBs titers. A decade later, she presented with ascites, peripheral edema, and hepatic dysfunction. Investigations revealed cirrhosis with ascites, markedly elevated aminotransferases, and new positivity for HBsAg and HBeAg, with an HBV DNA level of 797,000 IU/mL, despite no immunosuppression.

She was diagnosed with decompensated cirrhosis secondary to HBV reactivation with reverse seroconversion. Entecavir was initiated, resulting in clinical stabilization and a substantial decline in HBV DNA. This case underscores the potential for late spontaneous HBV reactivation despite prior anti-HBs, highlighting the importance of long-term monitoring in patients with past HBV exposure and HCV coinfection.

Several cases have documented spontaneous HBV reactivation in the absence of identifiable triggers. Our patient shared several features with previously reported cases including advanced age, no recent immunosuppression, and a long-standing resolved HBV infection with HBsAb positivity. Her case is unique in that there was no identifiable trigger, she had markedly high HBsAb, and she was otherwise healthy with minimal medical complexity compared to other reported cases.

**Conclusions:**

This presentation adds to the sparse literature that reverse HBV seroconversion can occur spontaneously in immunocompetent individuals, particularly the elderly. We suspect that many more cases have been unreported and perhaps even unrecognized. Clinicians should maintain long-term vigilance in patients with resolved HBV infection, especially those with prior HCV co-infection, as immune senescence or subtle physiologic stressors may precipitate reactivation even years after apparent viral control.

**Funding Agencies:**

None

